# Intralesional vitamin D versus triamcinolone acetonide for the treatment of keloids: a systematic review and meta-analysis of randomized controlled trials

**DOI:** 10.3389/fmed.2026.1823636

**Published:** 2026-06-03

**Authors:** Dan Jiang, Yalan Liu, Sheng Li, Ximao Wang, Shunjie You

**Affiliations:** 1 Department of Burn and Plastic Surgery, West China Hospital of Sichuan University-Ziyang Hospital, Ziyang Central Hospital, Ziyang, Sichuan, China; 2 Department of General Surgery, Sichuan Provincial People's Hospital East Sichuan Hospital & Dazhou First People's Hospital, Dazhou, Sichuan, China; 3 Dazhou Maternal and Child Health Hospital, Dazhou, Sichuan, China; 4 Department of Plastic and Burn Surgery, National Key Clinical Construction Specialty, The Affiliated Hospital of Southwest Medical University, Luzhou, Sichuan, China

**Keywords:** keloids, meta-analysis, systematic review, triamcinolone acetonide, vitamin D

## Abstract

Intralesional triamcinolone acetonide (TAC) remains the standard nonsurgical treatment for keloids but is limited by steroid-related adverse effects. Intralesional vitamin D has recently emerged as a potential alternative. This meta-analysis evaluated the comparative efficacy and safety of vitamin D versus TAC in keloid management. A systematic search of PubMed, Embase, and the Cochrane library identified randomized controlled trials comparing these treatments. The primary outcome was scar improvement assessed using validated scales, while secondary outcomes included lesion flattening and treatment-related adverse events. Four trials were included. TAC demonstrated superior efficacy in improving scar severity (MD = −9.72; 95% CI -17.41 to −2.02; *p* = 0.013) and lesion flattening (RR = 0.68; 95% CI 0.48 to 0.96; *p* = 0.028). Vitamin D was associated with significantly lower risks of pigmentation changes (RR = 0.23; 95% CI 0.07 to 0.78; *p* = 0.018) and skin atrophy (RR = 0.51; 95% CI 0.32 to 0.82; *p* = 0.006), with no significant differences in pain, erythema, telangiectasia, or blister formation. While TAC appeared more effective for scar regression and lesion flattening, vitamin D was associated with a more favorable safety profile, particularly regarding skin atrophy and dyspigmentation. Vitamin D may be considered in selected patients who are particularly concerned about steroid-related adverse effects or lesions located in cosmetically sensitive areas, although these findings should be interpreted cautiously given the limited evidence base.

## 1 Introduction

Keloids are benign fibroproliferative lesions that typically develop in susceptible individuals following skin trauma, such as burns, surgery, or inflammation. Although the precise pathophysiology remains unclear, the consensus is that keloids result from an abnormal wound healing process characterized by excessive fibroblast proliferation and collagen deposition (1, 2). Several factors contribute to this dysregulation, including anatomical location, wound depth, delayed healing, and skin tension, as they can trigger persistent inflammation and drive scar formation (3). While not life-threatening, keloids significantly impact a patient’s quality of life. Physically, they often cause pruritus, tenderness, pain, and restricted mobility. Psychologically, the cosmetic disfigurement, especially in prominent areas, can lead to distress and hinder social interactions. Functional limitations together with the psychological burden arising from treatment-related stress and dissatisfaction can substantially compromise patients’ quality of life (4, 5).

Keloid management remains challenging, with multiple therapeutic options available, including triamcinolone acetonide (TAC), botulinum toxin A, verapamil, 5-fluorouracil (5-FU), cryotherapy, and compression therapy (6). Among these, TAC is the most widely utilized therapy. TAC functions by suppressing the inflammatory response and inhibiting fibroblast proliferation. It also reduces collagen synthesis while increasing collagenase concentration, thereby facilitating the breakdown of excess scar tissue (7). This approach is proven to reduce scar volume and alleviate symptoms like itching and pain.

Vitamin D, a fat-soluble vitamin obtained through sunlight exposure, diet, or supplementation, has emerged as a potential therapeutic candidate. Beyond its classic role in calcium homeostasis, Vitamin D helps regulate cell differentiation, apoptosis, and proliferation. Crucially, it modulates genes involved in the epithelial-to-mesenchymal transition (EMT), a pathway central to fibrosis and scar formation (8). Alterations in vitamin D receptor (VDR) expression have been observed in keloid tissue, with reduced nuclear localization compared to normal skin (9). Consistent with this finding, emerging evidence indicates that vitamin D deficiency may increase the risk of keloid development, suggesting a potential role for vitamin D in scar modulation and management (10, 11).

Although recent studies have explored the therapeutic potential of intralesional vitamin D in the management of keloids, no clear consensus has yet been reached regarding its efficacy and safety. Consequently, the clinical value of vitamin D as an alternative to conventional therapies remains uncertain. Therefore, this study aims to systematically synthesize the currently available randomized evidence comparing vitamin D with triamcinolone acetonide (TAC) to provide more robust clinical evidence into their efficacy and safety in the treatment of keloids.

## 2 Methods

This systematic review and meta-analysis was conducted according to the Preferred PRISMA 2020 Statement (12). The study protocol was registered on the International PROSPERO, registration number: CRD420261308294. In this study, two authors independently searched databases, selected studies, extracted data, and assessed the risk of bias, while the third author resolved any disagreements that arose during the process independently.

### 2.1 Ethical statement

Since our meta-analysis was based on data from previously published studies, ethical approval and informed consents were not required.

### 2.2 Data sources and search strategy

A systematic literature search was conducted in three electronic databases: PubMed, Embase, and the Cochrane library. The search was performed on 24 December, 2025, using a pre-defined search strategy. The full search strategies for each database are detailed in Supplementary File 1. The reports were not limited to any language, with no publication date restrictions. Additionally, we performed a manual search of reference lists from key articles to identify other relevant studies.

### 2.3 Study selection criteria

Initially, the titles and abstracts of all articles retrieved from these databases were screened, and subsequently, a second screening was conducted for the full text. Literature selection was based on the following inclusion and exclusion criteria. The inclusion criteria: (1) studies that enrolled patients diagnosed with keloids; (2) studies comparing intralesional Vitamin D against TAC; (3) studies reporting quantitative data on treatment efficacy or safety outcome; (4) The study design was randomized controlled trials (RCT). The exclusion criteria: (1) non-original research, including reviews, meta-analyses, case reports, editorials, expert opinions, animal studies, and conference abstracts; (2) studies with insufficient data for analysis. (3) studies with duplicate patients.

The primary outcome was scar improvement, quantified as the change from baseline based on validated scar assessment scales. The treatment effect was estimated using the mean difference (MD) with 95% confidence intervals (CI). Secondary outcomes included the incidence of treatment-related local adverse events and clinical response events. These comprised skin atrophy, pigmentation changes, lesion flattening (defined as >50% reduction based on physician global assessment), pain, blister formation, telangiectasia, and erythema. Dichotomous outcomes were analyzed by calculating risk ratios (RR) with corresponding 95% CIs to assess differences between the vitamin D and TAC groups.

### 2.4 Data extraction and quality assessment

Using a predefined data extraction form, two independent reviewers extracted data on the following variables: (1) study characteristics (authors; country in which the study was performed; study design; follow-up time; scar evaluation scale; treatment protocol). (2) patient characteristics (sample size; age; sex; number of lesions treated).

The quality of the included studies was assessed using the Cochrane Risk of Bias 2 (RoB 2) tool for RCT (13). This tool evaluates five domains of potential bias, including bias arising from the randomization process, deviations from intended interventions, missing outcome data, measurement of the outcome, and selection of the reported results. Each domain was judged as having a low risk of bias, some concerns, or high risk of bias according to the RoB 2 guidance. An overall risk of bias judgment for each study was then determined based on these domain-level assessments. By taking into account bias risk, inconsistency, imprecision, indirectness, and publication bias, the Grading of Recommendations Assessment, Development, and Evaluation (GRADE) technique was used to rate the certainty of the evidence. The assessment was performed independently by two reviewers, and any disagreements were resolved by consensus or by involving a third author.

### 2.5 Statistical analysis

For the primary outcome, the pooled effect was calculated based on the change from baseline in validated scar assessment scales. For secondary outcomes, RR with 95% CI were calculated based on the number of events and the total number of lesions or participants in each group. Effect estimates were extracted directly from the included studies where available or calculated from the reported data when necessary. Due to the limited number of included study, we will not do subgroup analysis. Given the potential heterogeneity in study settings, we use random effects model for analysis. The degree of heterogeneity was assessed using the I^2^ test, with values < 30%, 30–50%, and > 50% indicating low, moderate, and high levels of heterogeneity, respectively. We performed a sensitivity analysis to assess the robustness of our analysis. Due to the limited number of included studies, funnel plot analysis and Begg’s test were not performed. The Stata 18 (StataCorp, College Station, TX, USA) software were used to conduct all statistical analyses. *p* < 0.05 was considered to be statistically significant.

## 3 Results

### 3.1 Study selection and characteristics

**Figure 1:**
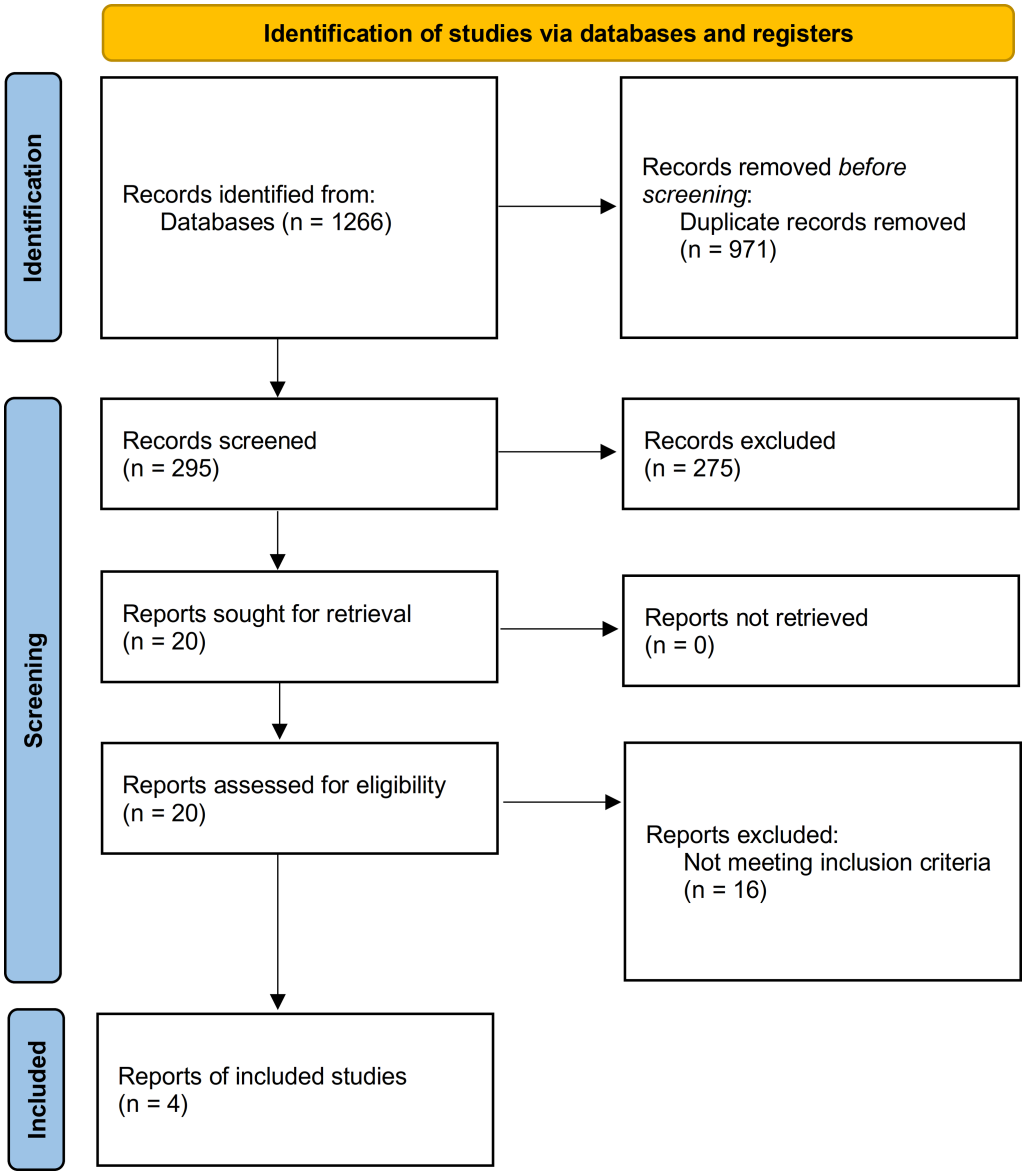
RISMA flow chart of the study selection process.

The study selection process is shown on the PRISMA flow chart (Figure 1). A total of 1,266 articles were initially identified; 971 articles removed because of duplicates. Following title and abstract screening based on predefined inclusion and exclusion criteria, 275 articles were excluded. We conducted a full-text assessment of the remaining 20 studies, and 16 were excluded. Finally, 4 studies involving a total of 121 patients were considered eligible and included in the meta-analysis (14–17). The baseline characteristics of the included studies are summarized in Table 1. All included articles were RCT published between 2024 and 2025. The mean age of participants varied across studies, ranging from 26.34 to 35.23 years, and the male populations were ranging from 50.0 to 55.6%. The follow-up periods ranging from 4 to 12 weeks. All included studies used the Vancouver Scar Scale (VSS) to assess scar outcomes.

**Table 1:** Baseline characteristics of the included studies.

Study	Country	Study design	Age (mean)	Sample Size (n)	Sex (M/F)	Lesions treated (n)	Intervention protocol (Vitamin D)	Control protocol (TAC)	Scar evaluation scale	Follow-up (weeks)
Pazyar et al. (2024) (17)	Iran	RCT	35.23 ± 8.57	22	12/10	44	300,000 IU, q3w	20 mg/mL, q3w	VSS	12
Goyal et al. (2024) (15)	India	RCT	33.00 ± 10.78	60	31/29	60	200,000 IU, q4w	20 mg/ml, q4w	VSS	8
Galal et al. (2025) (14)	Egypt	RCT	26.34 ± 6.76	30	15/15	30	200,000 IU, q3w	10 mg/mL, q3w	VSS	12
Mohapatra et al. (2024) (16)	India	RCT	NR	9	5/4	60	60,000 IU, q4w	40 mg/mL, q4w	VSS	4

RCT, randomized controlled trial; M/F, male/female; IU, international units; q3w, every 3 weeks; q4w, every 4 weeks; TAC, triamcinolone acetonide; VSS, Vancouver Scar Scale; NR, not reported.

**Figure 2:**
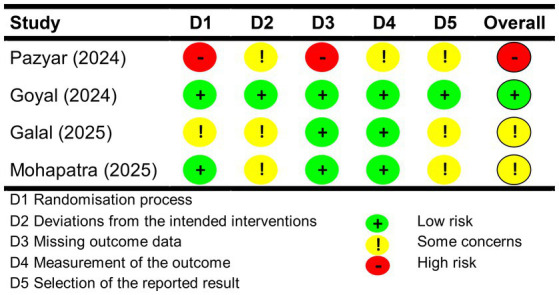
Risk of bias assessment of the included randomized controlled trials using the Cochrane RoB 2 tool across five domains: D1, randomization process; D2, deviations from intended interventions; D3, missing outcome data; D4, measurement of the outcome; and D5, selection of the reported result.

The methodological quality of the four included trials was assessed using the RoB2. Overall, the quality of the evidence was considered acceptable. Two studies were assessed as having a low risk of bias, one study was judged to have some concerns, and one study was rated as having a high risk of bias. A summary of the risk of bias assessment for each included study is provided in Figure 2 and Supplementary File 2. The GRADE assessment of the included studies showed that the certainty of evidence ranged from moderate to low across the evaluated outcomes (Supplementary File 3). Overall, the certainty of evidence was rated as moderate for scar improvement, lesion flattening, pigmentation changes, skin atrophy, and telangiectasia. In contrast, the certainty of evidence was judged as low for pain, erythema, and blister formation. The main reasons for downgrading the certainty of evidence were inconsistency and imprecision, particularly for outcomes with a limited number of studies, small sample sizes, or wide confidence intervals. No serious concerns were identified regarding risk of bias, indirectness, or publication bias. Overall, these findings suggest that the current evidence provides moderate certainty for most efficacy and safety outcomes, although additional high-quality studies are still needed to improve precision and strengthen confidence in several adverse-event outcomes.

### 3.2 Efficacy and safety outcomes

Skin atrophy, blister formation, lesion flattening, and telangiectasia showed low heterogeneity (I^2^ = 0%). In contrast, scar improvement (I^2^ = 59.87%), pigmentation changes (I^2^ = 34.24%), pain (I^2^ = 50.26%), and erythema (I^2^ = 67.22%) demonstrated moderate to substantial heterogeneity.

**Figure 3:**
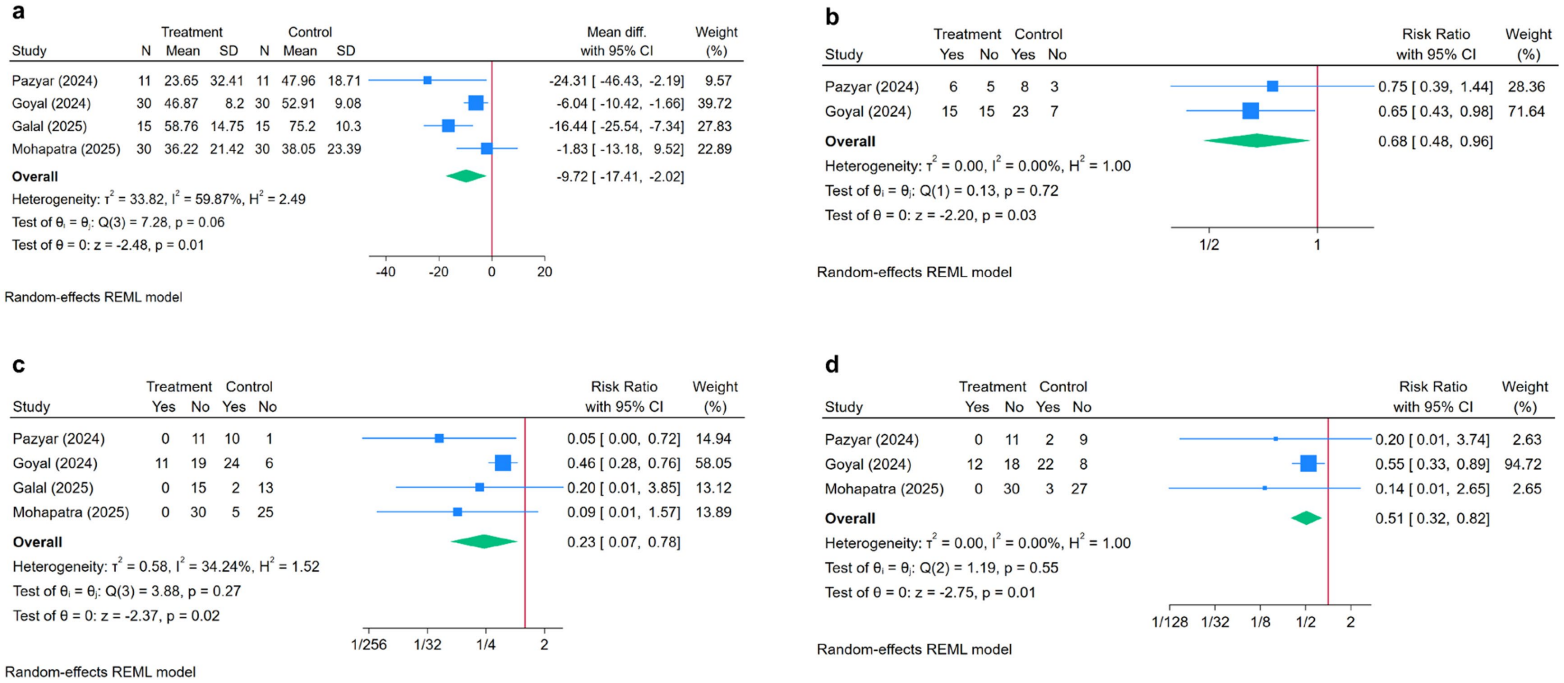
Forest plots comparing intralesional vitamin D with triamcinolone acetonide for selected clinical outcomes: **(a)** scar improvement; **(b)** lesion flattening; **(c)** pigmentation changes; **(d)** skin atrophy.

For the primary outcome, four RCT were included in the analysis of scar improvement. Compared with TAC, intralesional vitamin D showed a significantly lower degree of scar improvement (MD = −9.72; 95% CI -17.41 to −2.02; *p* = 0.013; Figure 3a). Similarly, lesion flattening, reported in two studies, was significantly less likely to occur in the vitamin D group (RR = 0.68; 95% CI 0.48 to 0.96; *p* = 0.028; Figure 3b). Four studies contributed to the analysis of pigmentation changes, which were significantly less frequent in the vitamin D group compared with TAC (RR = 0.23; 95% CI 0.07 to 0.78; *p* = 0.018; Figure 3c). In terms of adverse events, three studies reported skin atrophy, which occurred significantly less frequently in the vitamin D group than in the TAC group (RR = 0.51; 95% CI 0.32 to 0.82; *p* = 0.006; Figure 3d).

**Figure 4:**
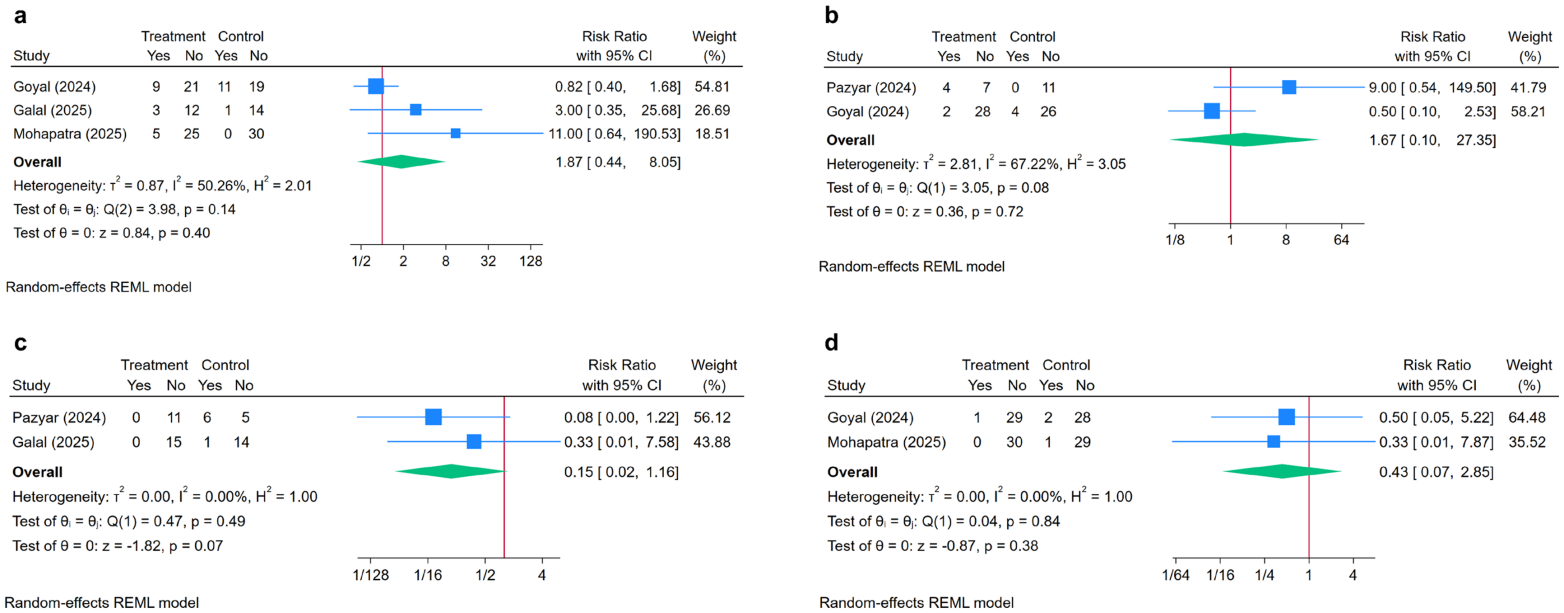
Forest plots comparing intralesional vitamin D with triamcinolone acetonide for selected clinical outcomes: **(a)** pain; **(b)** erythema; **(c)** telangiectasia; **(d)** blister formation.

Pain was reported in three studies and showed no significant difference between the vitamin D and TAC groups (RR = 1.87; 95% CI 0.44 to 8.05; *p* = 0.437; Figure 4a). Similarly, erythema and telangiectasia, each reported in two studies, did not differ significantly between groups (RR = 1.67; 95% CI 0.10 to 27.35; *p* = 0.0718; Figure 4b and RR = 0.15; 95% CI 0.02 to 1.16; *p* = 0.069; Figure 4c, respectively). Blister formation was also comparable between the two groups based on two studies (RR = 0.43; 95% CI 0.07 to 2.85; *p* = 0.384; Figure 4d). All the forest plot for outcomes are shown in Figures 3, 4.

### 3.3 Sensitivity analysis

To assess the stability of our findings, a leave-one-out sensitivity analysis was conducted. For the primary outcome of scar improvement, the results were robust. Omitting any individual study did not alter the statistical significance of the pooled estimate, confirming the stability of the observed treatment effect. For secondary outcomes, the pooled effects for skin atrophy and lesion flattening were partially influenced by the inclusion of individual studies, with changes in statistical significance observed in leave-one-out analyses. For other outcomes, including pigmentation, erythema, telangiectasia, blistering, and pain, the results remained generally stable. All the results for sensitivity analysis are shown in Supplementary File 4.

## 4 Discussion

Our systematic review and meta-analysis, synthesizing data from four RCT, provides the most comprehensive evidence to date regarding the efficacy and safety of intralesional vitamin D for the treatment of keloids. Overall, TAC remains more effective in achieving clinical scar regression, and vitamin D was less effective in promoting lesion flattening, further supporting the superior anti-fibrotic efficacy of TAC. However, vitamin D was associated with a more favorable safety profile, particularly showing a reduced risk of pigmentation changes and skin atrophy. Nevertheless, for several other adverse outcomes, such as pain, erythema, telangiectasia, and blister formation, no significant differences were observed between the two treatments.

The observed results appear biologically plausible when viewed through the dominant pathophysiology of keloids. Keloids are fibroproliferative scars characterized by persistent inflammation and dysregulated extracellular matrix (ECM) remodeling, which promote fibroblast proliferation, myofibroblast differentiation, and excessive collagen deposition (18). Intralesional TAC delivers a high local concentration of glucocorticoid that can suppress inflammatory processes, reduce collagen and glycosaminoglycan synthesis, inhibit fibroblast proliferation, and influence key growth-factor pathways (19).

In contrast, vitamin D exerts anti-fibrotic effects primarily through activation of the VDR. VDR signaling has been shown to reduce collagen type I, fibronectin, and *α*-smooth muscle actin expression in keloid fibroblasts, while modulating ECM remodeling and promoting hepatocyte growth factor production (9). However, its clinical effectiveness may be limited by translational factors. Many studies use cholecalciferol rather than the active form, making local efficacy dependent on tissue conversion. Cutaneous vitamin D metabolism is more active in epidermal keratinocytes than in dermal fibroblasts, which may have a limited capacity for full activation of vitamin D in scar tissue (20). In addition, reduced VDR protein levels and/or reduced nuclear localization has been reported in keloid tissue versus normal skin, which could theoretically reduce responsiveness to vitamin D–based interventions (21). Taken together, these pharmacologic and tissue-level considerations collectively offer a coherent explanation for why intralesional vitamin D might improve certain clinical parameters but show comparatively weaker effects on lesion flattening than TAC.

Across existing keloid management, intralesional TAC remains regarded as a core first-line nonsurgical option, supported by prior evidence demonstrating meaningful reductions in scar size and symptoms (18). Our findings align with this position by confirming the superior efficacy of TAC in achieving greater scar regression and lesion flattening compared with vitamin D.

Accordingly, from a clinical decision-making perspective, TAC may remain the preferred option when rapid and substantial lesion flattening or regression is the primary treatment objective. However, intralesional vitamin D demonstrated a more favorable safety profile, particularly with a lower risk of pigmentary changes and skin atrophy. This suggests an important role of vitamin D in selected patients who are more concerned about steroid-induced atrophy, cosmetically sensitive treatment sites, or dyspigmentation. Current data also imply that vitamin D may have a slower onset of benefit than TAC, so patient counseling should explicitly address expectations regarding response kinetics and the potential need for more sessions or longer follow-up to appreciate maximal improvement (16, 17). In clinical practice, keloid management is often individualized and multimodal. From a clinical decision-making perspective, the present findings suggest that TAC may remain the preferred option when rapid lesion flattening or regression is the primary therapeutic goal. In contrast, intralesional vitamin D may be considered as an alternative option for patients who are particularly concerned about steroid-related adverse effects, cumulative steroid exposure, or lesions located in cosmetically sensitive areas. Based on the present findings, when rapid lesion flattening or regression is the main therapeutic objective, TAC may be used initially and then switched to vitamin D after a clear therapeutic response has been achieved, with the aim of reducing steroid-related adverse effects; such an approach does exist in clinical practice and appears to be reasonable. However, because direct evidence evaluating TAC–vitamin D sequential or alternating treatment strategies remains very limited, we were unable to identify clear evidence showing that TAC–vitamin D sequential or alternating strategies are more effective. Moreover, the studies included in our meta-analysis compared these two agents head-to-head rather than evaluating them as part of a defined treatment sequence.

In theory, intralesional vitamin D may be associated with injection-site pain, which is likely related to the oil-based formulations. These preparations are absorbed and diffuse slowly within subcutaneous tissues, potentially generating local mechanical tension and stimulating sensory nerve endings, leading to symptoms such as stinging, burning, or aching sensations. In our analysis, although a higher proportion of patients in the vitamin D group reported pain, the difference did not reach statistical significance. This may be attributed to the limited sample size and variability in injection techniques, and further research is warranted to better clarify this observation.

Several limitations of this meta-analysis should be acknowledged. First, the number of included trials was limited, with relatively small sample sizes, which may have reduced the statistical power of some outcomes. Second, there was potential clinical heterogeneity in the intervention protocols across studies. Variations in the concentration of vitamin D, injection frequency, duration of therapy, and TAC concentration may have contributed to differences in treatment response and adverse event rates. Third, because of the limited number of eligible studies, we were unable to perform subgroup analyses to further explore potential sources of heterogeneity. Fourth, scar formation and recurrence are influenced by local skin tension, which varies by anatomical site and may affect treatment response. The lack of detailed reporting on lesion location in some included studies may have introduced potential bias into the pooled estimates. Finally, the follow-up duration in most included studies was relatively short. Given the high recurrence rate associated with keloids, the current evidence may not fully capture the long-term efficacy of vitamin D in preventing scar recurrence.

## 5 Conclusion

In conclusion, compared with intralesional vitamin D, TAC appeared more effective for scar regression and lesion flattening in patients with keloids, whereas vitamin D was associated with lower risks of skin atrophy and dyspigmentation. These findings suggest that intralesional vitamin D may represent a potential option in selected patients who are particularly concerned about steroid-related adverse effects or lesions located in cosmetically sensitive areas. However, given the limited number of randomized controlled trials, relatively small sample sizes, and short follow-up duration, these results should be interpreted cautiously. Future large-scale, high-quality RCTs with longer follow-up are warranted to clarify long-term outcomes, including recurrence, and to further define the role of vitamin D in keloid management.

## Data Availability

The original contributions presented in the study are included in the article/Supplementary material, further inquiries can be directed to the corresponding author.
