# Supplementary material for: Intralesional vitamin D versus triamcinolone acetonide for the treatment of keloids: a systematic review and meta-analysis of randomized controlled trials
Source: Front Med (Lausanne). 2026 Jun 3;13:1823636. doi: 10.3389/fmed.2026.1823636 (PMC13272075; doi:10.3389/fmed.2026.1823636)
Supplement: Supplementary file 1 [file Data_Sheet_1.DOCX]

Supplementary Material

# Supplementary File 1. Detailed Search Strategy.

Source: PubMed

Searched on: 2025.12.24

Results: 116

| Search | Query | Results |
| --- | --- | --- |
| #1 | ((cicatrix, hypertrophic[MeSH Terms]) OR keloid[MeSH Terms]) OR hypertrophy[MeSH Terms] | 89626 |
| #2 | (((keloid*[Title/Abstract]) OR hypertrophic[Title/Abstract]) OR cicatrix[Title/Abstract]) OR scar*[Title/Abstract] | 318349 |
| #3 | #1 OR #2 | 390557 |
| #4 | Vitamin D [MeSH Terms] | 73599 |
| #5 | ((((((((((((Vitamin D [Title/Abstract]) OR Vitamin D group [Title/Abstract]) OR Group of vitamin D compounds [Title/Abstract] OR Vitamin D compounds [Title/Abstract]OR Vitamin D analogues [Title/Abstract]) OR Vitamin D hormone system [Title/Abstract]) OR Vitamin D3 [Title/Abstract]) OR Sunshine vitamin [Title/Abstract]) OR Antirachitic vitamin [Title/Abstract]) OR Vit D [Title/Abstract]) OR Vit. D [Title/Abstract]) OR 25(OH)D [Title/Abstract]) OR Cholecalciferol [Title/Abstract]) OR 1α-hydroxyvitamin D3 [Title/Abstract]) | 26301 |
| #6 | #4 OR #5 | 79748 |
| #7 | ((((((randomly[Title/Abstract]) OR trial[Title])) OR ((((randomized controlled trial[Publication Type]) OR controlled clinical trial[Publication Type]) OR randomized[Title/Abstract]) OR placebo[Title/Abstract])) OR "Clinical Trials as Topic"[Mesh:NoExp])) NOT ((animals[MeSH Terms]) NOT ((humans[MeSH Terms]) AND animals[MeSH Terms])) | 1620637 |
| #8 | #3 AND #6 AND #7 | 116 |

Source: EMBASE

Searched on: 2025.12.24

Results: 932

| Search | Query | Results |
| --- | --- | --- |
| #1 | 'hypertrophic scar'/exp OR 'keloid'/exp OR 'hypertrophy'/exp | 470574 |
| #2 | keloid*:ab,ti OR hypertrophic:ab,ti OR cicatrix:ab,ti OR scar*:ab,ti | 434932 |
| #3 | #1 OR #2 | 775943 |
| #4 | ' vitamin D '/exp | 211139 |
| #5 | vitamin D:ab,ti OR vitamin D group:ab,ti OR group of vitamin D compounds:ab,ti OR group of vitamin D compounds:ab,ti OR vitamin D analogues:ab,ti OR vitamin D hormone system:ab,ti OR vitamin D3:ab,ti OR sunshine vitamin:ab,ti OR antirachitic vitamin:ab,ti OR vit D:ab,ti OR vit. D:ab,ti OR cholecalciferol:ab,ti OR 1α-hydroxyvitamin D3:ab,ti | 12418 |
| #6 | #4 OR #5 | 211788 |
| #7 | 'crossover procedure':de OR 'double-blind procedure':de OR 'randomized controlled trial':de OR 'single-blind procedure':de OR random*:de,ab,ti OR factorial*:de,ab,ti OR crossover*:de,ab,ti OR ((cross NEXT/1 over*):de,ab,ti) OR placebo*:de,ab,ti OR ((doubl* NEAR/1 blind*):de,ab,ti) OR ((singl* NEAR/1 blind*):de,ab,ti) OR assign*:de,ab,ti OR allocat*:de,ab,ti OR volunteer*:de,ab,ti | 3974319 |
| #8 | #3 AND #6 AND #7 | 932 |
|  |  |  |

Source: Cochrane library

Searched on: 2025.12.24

Results: 29

| Search | Query | Results |
| --- | --- | --- |
| #1 | ((cicatrix, hypertrophic[MeSH Terms]) OR keloid[MeSH Terms]) OR hypertrophy[MeSH Terms] | 9212 |
| #2 | (keloid):ti,ab,kw OR (hypertrophic):ti,ab,kw OR (cicatrix):ti,ab,kw OR (scar):ti,ab,kw | 9508 |
| #3 | #1 OR #2 | 17264 |
| #4 | Vitamin D [MeSH Terms] | 21692 |
| #5 | (vitamin D):ti,ab,kw OR (vitamin D group):ti,ab,kw OR (group of vitamin D compounds):ti,ab,kw OR (group of vitamin D compounds):ti,ab,kw OR (vitamin D analogues):ti,ab,kw OR (vitamin D hormone system):ti,ab,kw OR (vitamin D3):ti,ab,kw OR (sunshine vitamin):ti,ab,kw OR (antirachitic vitamin):ti,ab,kw OR (vit D):ti,ab,kw OR (vit. D):ti,ab,kw OR (cholecalciferol):ti,ab,kw OR (1α-hydroxyvitamin D3):ti,ab,kw OR (25(OH)D):ti,ab,kw | 20073 |
| #6 | #4 OR #5 | 23050 |
| #7 | #3 AND #6 | 178 |
| #8 | trials | 2338047 |
| #9 | #7 AND #8 | 178 |

**Supplementary File 2**


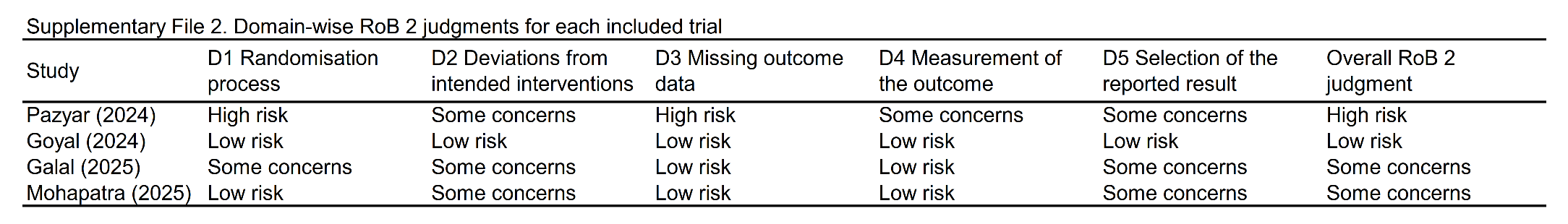


**Supplementary File 3**


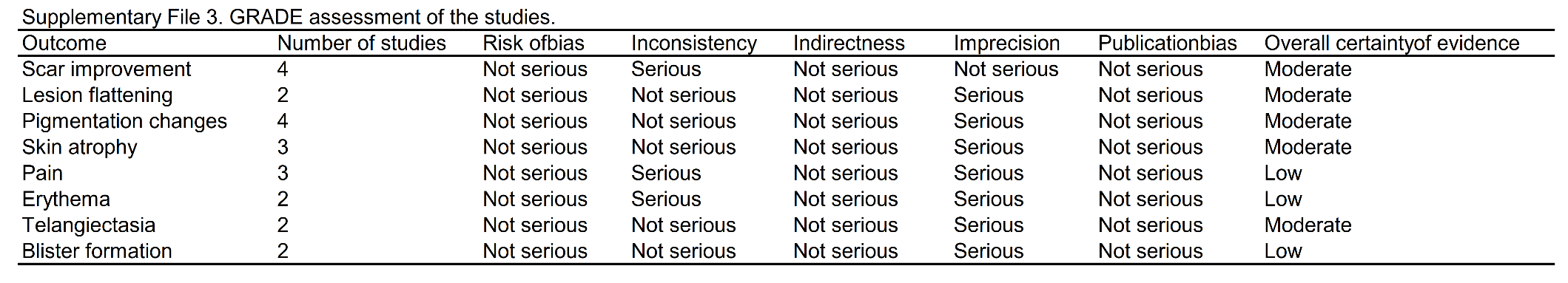


**Supplementary File 4.** Sensitivity Analysis.

**
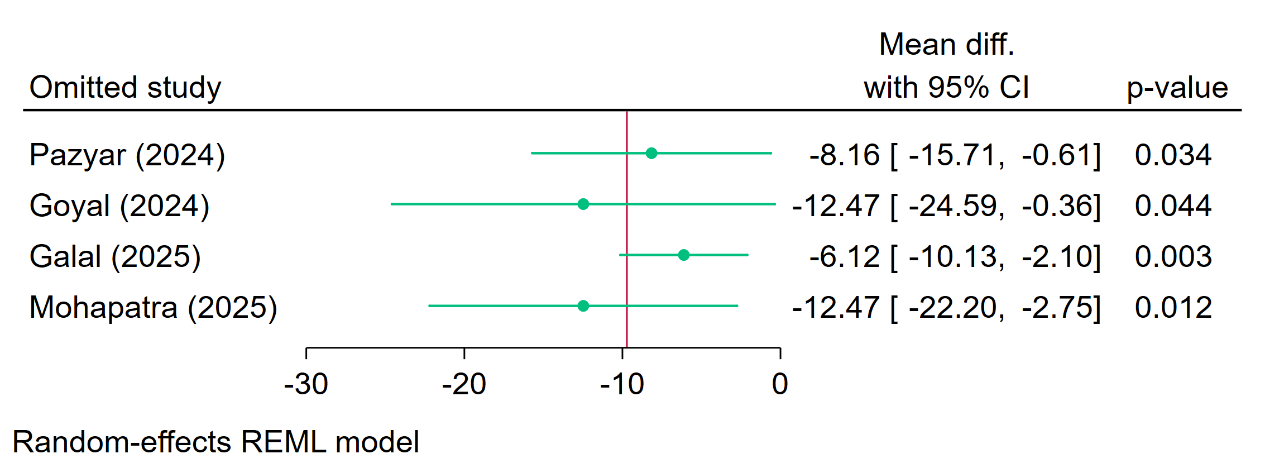
**

a. Sensitivity Analysis for scar improvement

**
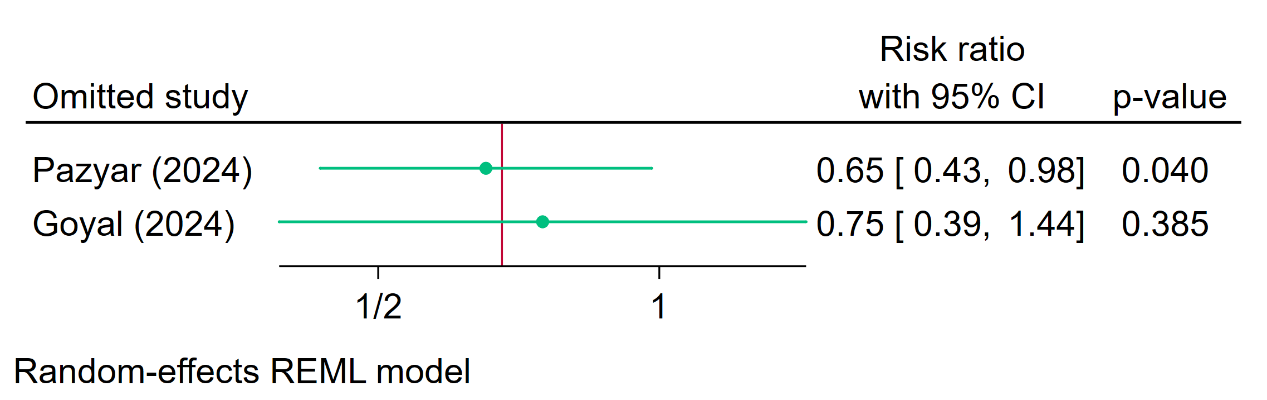
**

b. Sensitivity Analysis for lesion flattening

**
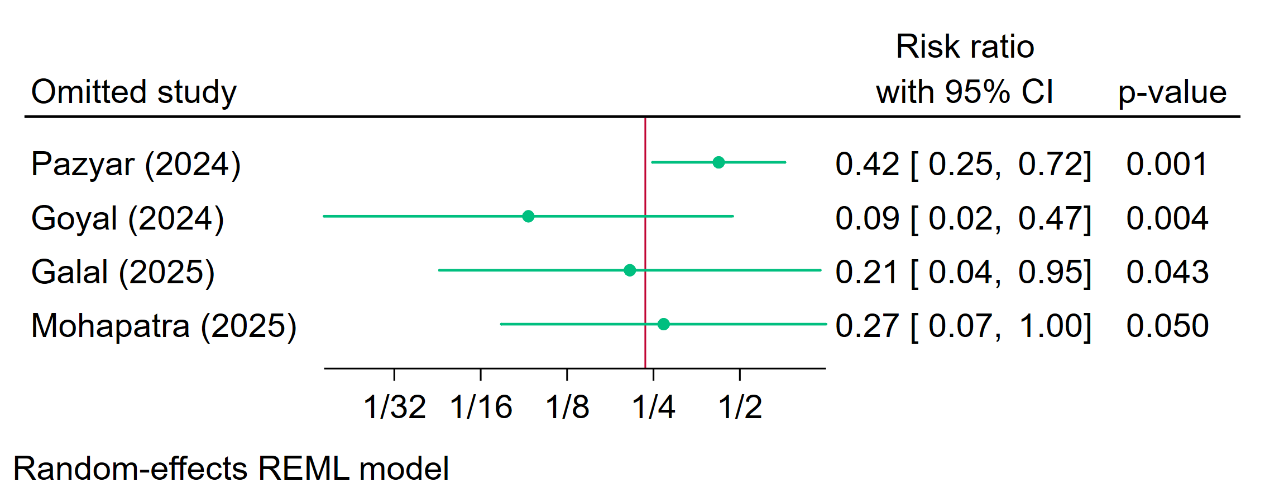
**

c. Sensitivity Analysis for pigmentation changes

**
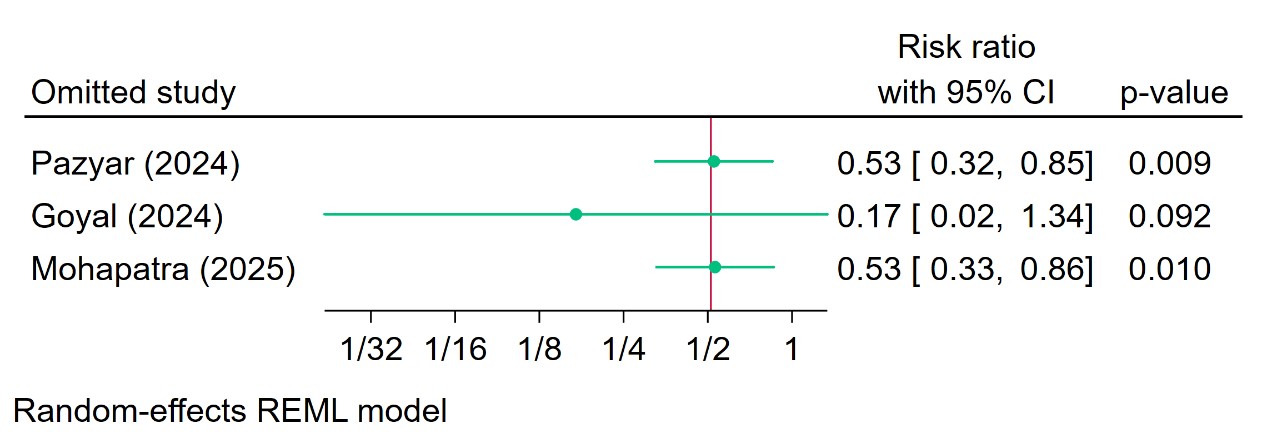
**

d. Sensitivity Analysis for skin atrophy

**
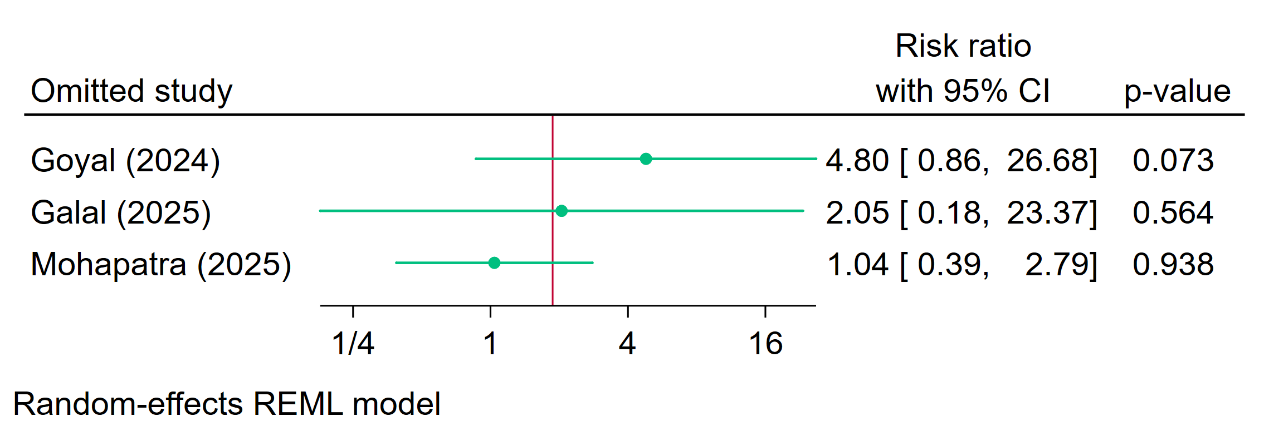
**

e. Sensitivity Analysis for Pain

**
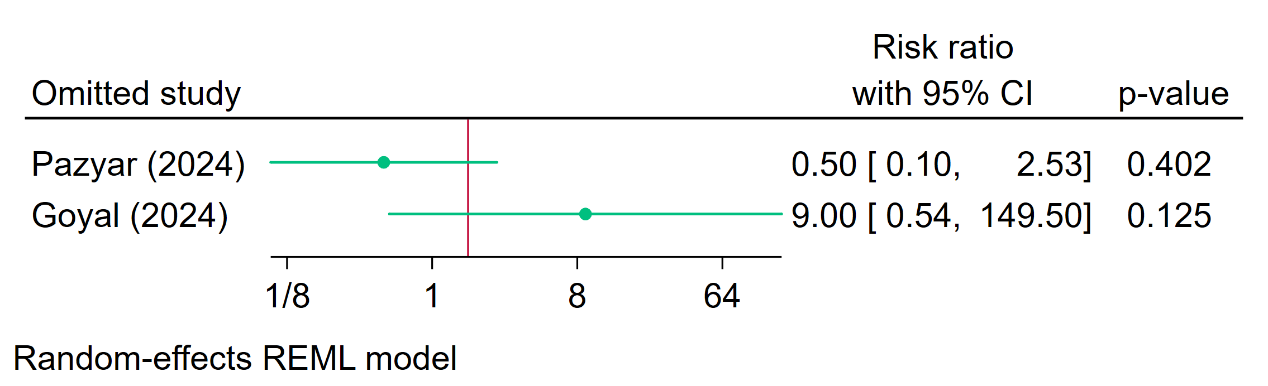
**

f. Sensitivity Analysis for erythema

**
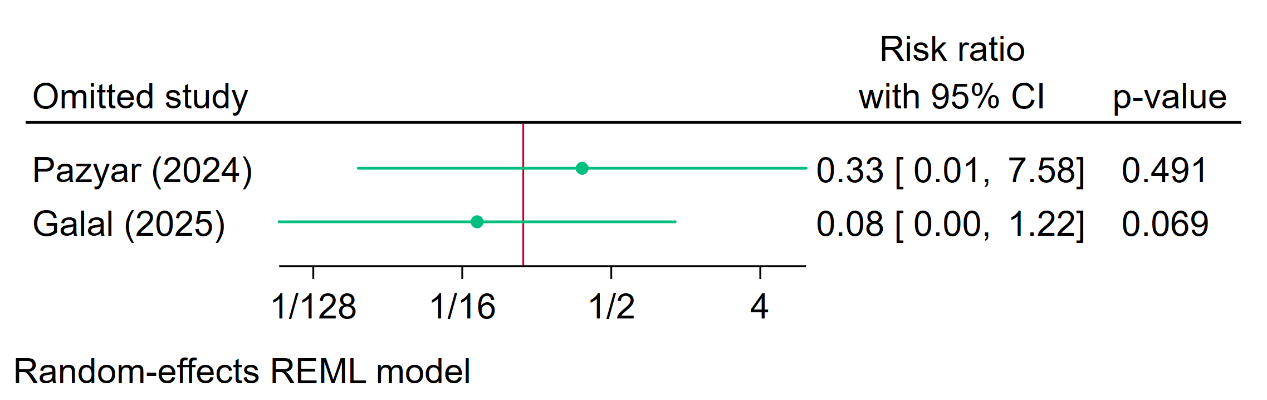
**

g. Sensitivity Analysis for telangiectasia

**
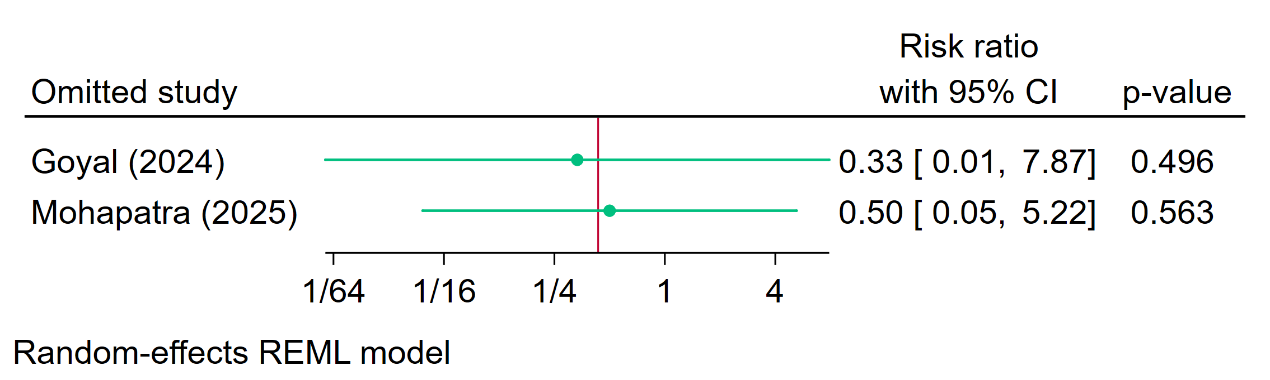
**

h. Sensitivity Analysis for Blister formation
